# Circulating Biomarkers in Long-Term Stroke Prognosis: A Scoping Review Focusing on the South African Setting

**DOI:** 10.7759/cureus.23971

**Published:** 2022-04-09

**Authors:** Juan Jansen van Vuuren, Somasundram Pillay, Ansuya Naidoo

**Affiliations:** 1 Department of Neurology, Grey’s Hospital, Pietermaritzburg, ZAF; 2 School of Clinical Medicine, PhD programme, University of KwaZulu-Natal, Pietermaritzburg, ZAF; 3 Member, Royal Society of South Africa, Cape Town, ZAF; 4 Clinical Medicine, University of KwaZulu-Natal, Durban, ZAF; 5 Neurology, University of KwaZulu-Natal, Pietermaritzburg, ZAF; 6 Department of Neurology, Grey's Hospital, University of KwaZulu-Natal, Pietermaritzburg, ZAF

**Keywords:** mortality, morbidity, biomarkers, south africa, stroke, prognosis

## Abstract

Cerebrovascular disease, including both ischaemic and haemorrhagic strokes, remains one of the highest causes of global morbidity and mortality. Developing nations, such as South Africa (SA), are affected disproportionately. Early identification of stroke patients at risk of poor clinical prognosis may result in improved outcomes. In addition to conventional neuroimaging, the role of predictive biomarkers has been shown to be important. Little data exist on their applicability within SA. This scoping review aimed to evaluate the currently available data pertaining to blood biomarkers that aid in the long-term prognostication of patients following stroke and its potential application in the South African setting.

This scoping review followed a 6-stage process to identify and critically review currently available literature pertaining to prognostic biomarkers in stroke.

An initial 1191 articles were identified and, following rigorous review, 41 articles were included for the purposes of the scoping review. A number of potential biomarkers were identified and grouped according to the function or origin of the marker. Although most biomarkers showed great prognostic potential, the cost and availability will likely limit their application within SA.

The burden of stroke is increasing worldwide and appears to be affecting developing countries disproportionately. Access to neuroradiological services is not readily available in all settings and the addition of biomarkers to assist in the long-term prognostication of patients following a stroke can be of great clinical value. The cost and availability of many of the reviewed biomarkers will likely hinder their use in the South African setting.

## Introduction and background

Cerebrovascular disease, including ischaemic and haemorrhagic strokes, remains one of the highest causes of morbidity and mortality globally, with an estimated stroke prevalence of 104.2 million people, resulting in over 6 million deaths in 2017 alone [1]. Low-to-middle income countries (LMIC), such as South Africa (SA), are disproportionately affected compared to wealthier nations [2,3]. Strokes result in an excess of 100 disability-adjusted life years (DALYs) in SA alone, with one study in rural SA showing a burden of the cost of more than R4.2-million (approximately US$264000) in sub-district health expenditure [4,5].

Early identification, confirmation and management of suspected acute ischaemic strokes (AIS) and intracerebral haemorrhages (ICH) result in improved functional outcomes [6,7]. Neuroradiological imaging, the gold standard diagnostic test in stroke medicine, incurs significant costs, with the global computed tomography (CT) scan market alone exceeding US$6billion in 2020 [8]. CT brain, the investigation of choice when a stroke is suspected, can cost upwards of ZAR4000 (approximately US$280), whilst magnetic resonance imaging (MRI) can cost more than ZAR10000 (approximately US$700). This clearly limits the wide implementation of these imaging modalities in LMIC, such as SA. The need for cheap, widely accessible diagnostic and prognostic tools such as biomarkers, which have been shown to have significant additive predictive value, is therefore evident [9].

The term biological marker, or biomarker, has been ascribed various definitions. Strimbu and Tavel and Puntmann emphasise the objectivity of measured biomarkers [10,11]. The Food and Drug Administration-National Institutes of Health (FDA-NIH) Biomarker Working Group published an updated version of their document titled BEST (Biomarkers, EndpointS, and other Tools) Resource on the 25th of January 2021. Various types of biomarkers are defined and discussed [12]. Of interest in the current review are prognostic biomarkers in acute strokes. A prognostic biomarker is “used to identify (the) likelihood of a clinical event, disease recurrence or progression in patients” presenting with a specific diagnosis [12].

A biomarker aiming to assist in long-term prognostication in strokes should ideally be detectable early in the disease process, widely available, easily interpreted, and must have an appropriate sensitivity to ensure false negatives are avoided. The use of biomarkers following various insults to the brain has been well described. Numerous studies and systematic reviews have been published over the years, including a study by Jickling and Sharp, who found that more than 58 biomarkers in ischaemic stroke have been described [13-19].

This scoping review aimed to determine the currently available data pertaining to blood biomarkers that aid in the long-term prognostication of patients following AIS or ICH and its application within the SA setting.

## Review

Although no definition of a scoping review has been universally accepted, essential themes and purposes have been identified which are ever-expanding [20,21]. Scoping reviews aim to rapidly determine important ideas in a specific research area [22]. It assists in determining the scope of research done in its breadth, depth and nature [22,23]. Mays et al. are of the view that scoping reviews “can be undertaken as standalone projects in their own right” [24]. Extensive work has been published by Arksey and O’Malley, further strengthened by work by Levac et al. [20,22]. These authors recommend a 6-stage process that has been adapted for the purpose of this review.

Stage 1 required establishing the research question and discussion with a team of researchers with expertise in the field. The question “What is the currently available literature regarding the evidence for the use of blood biomarkers in long-term prognostication following stroke?” was decided upon. A long-term prognosis is defined as functional or clinical outcome more than 30 days following the stroke event. Identification of relevant studies (stage 2) required the determination of keywords, which, with the help of Boolean operators (AND/OR/NOT), aided in refining the search terms in multiple databases. Following the application of filters such as English language and human participants, the search resulted in the identification of literature from PubMed/Medline (228 articles), ScienceDirect (890 articles) and SciELO (16 articles). A further 57 articles were identified during the reading process.

**Figure 1:**
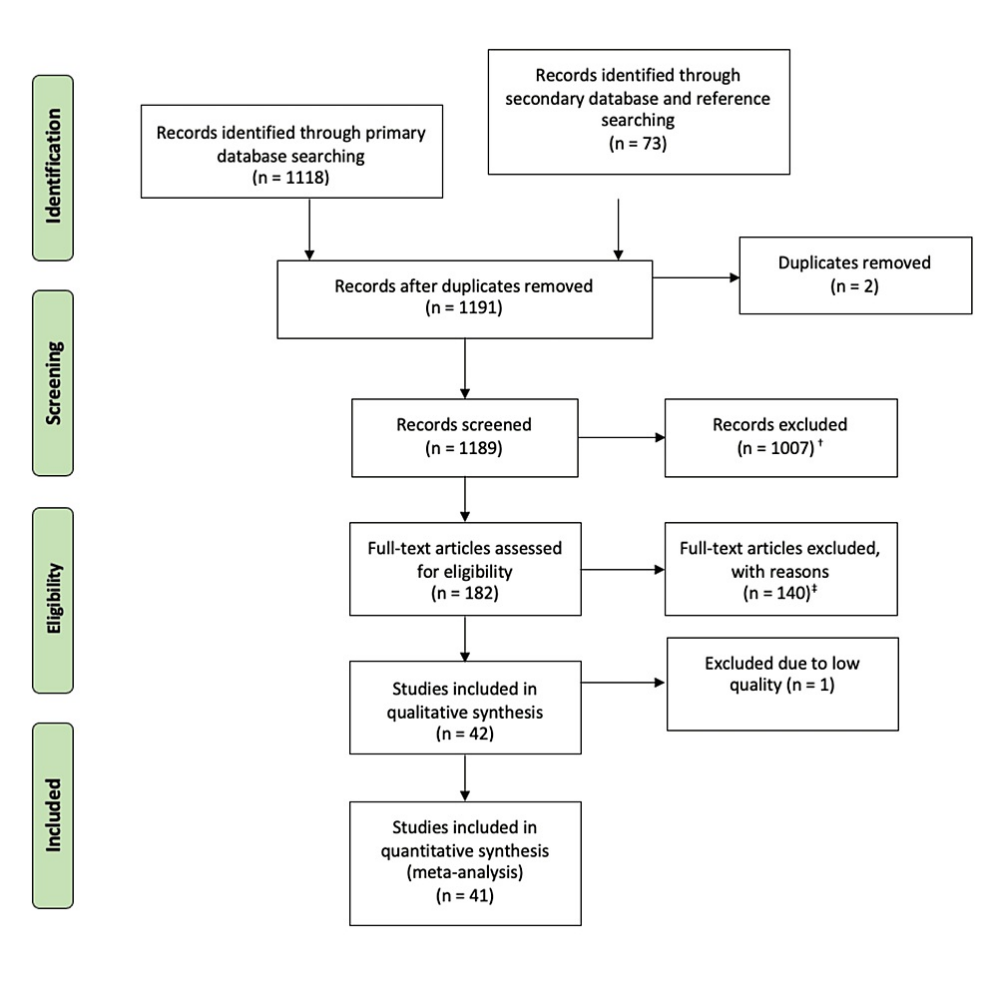
The Study Selection Process Following the PRISMA Flowchart PRISMA: Preferred Reporting Items for Systematic Reviews and Meta-Analysis. Original image created by the authors. ^†^Study/trial unrelated to research question: 583; study/trial focussing on stroke risk factors: 36; study/trial focussing on management of stroke: 24; study/trial focussing on cardiac pathologies: 283; study/trial focussing on atrial fibrillation: 35; study/trial focussing on cardiorenal syndromes: 5; study/trial focussing on coronavirus disease 2019 (COVID-19): 30; study/trial focussing on sickle cell disease: 8; study/trial focussing on artificial intelligence in disease: 3 ^‡^Review article/meta-analysis/editorial: 34; full text not available, only abstract: 6; study on stroke risk/chronic strokes/transient ischaemic attacks: 15; study on biomarkers and mainly other diseases in stroke: 20; study on differentiating stroke types and stroke mimics: 7; study focussing on diagnosis/management/clinical decision-making: 28; study determining risk of acute outcomes: 9; study to determine pathophysiology of disease: 7; study criteria not specific: 2; animal/laboratory-based studies (validation): 11; study looking at cost associated with stroke: 1

As a result, a total of 1191 articles were identified for analysis. The Preferred Reporting Items for Systematic Reviews and Meta-Analyses (PRISMA) flowchart was utilised to screen these articles for selection (stage 3) and, following the application of rigorous exclusion criteria, a total of 41 articles were included for critical review (Figure 1) [25].

Data were extracted and charted (stage 4) from each study following critical analysis based on the methods described by Young and Solomon [26]. Data were collated and summarised (stage 5) below, following which a conclusion was drawn (stage 6). Arksey and O’Malley recommend that “a thematic construction is used to provide an overview of the breadth of the literature” [22]. As such, following critical analysis, the 41 key articles have been summarised (Table 1) [27-67]. The nature of the articles has allowed for a categorical theme which is discussed below.

**Table 1:** Studies Included in the Scoping Review and the Biomarkers Assessed TNFa: tumour necrosis factor alpha; IL-1b: interleukin 1 beta; IL-1RA: interleukin 1 receptor antagonist; IL-6: interleukin 6; IL-10: interleukin 10; TNF-R1: tumour necrosis factor receptor 1; TNF-R2: tumour necrosis factor receptor 2; ICAM-1: intercellular adhesion molecules 1; ALCAM: activated leukocyte adhesion molecule; TBARS: thiobarbituric acid-reactive substances; Tim-3: T-cell immunoglobulin and mucin-domain 3; OPN: osteopontin; CRP: C-reactive protein; SAA: serum amyloid A; TM: thrombomodulin; FVIII: factor eight; beta-TG: beta-thromboglobulin; vWF: von Willebrand factor; t-PA: tissue plasminogen activator; FXIII A subunit: factor thirteen A subunit; ADAMTS13Ac: activity of disintegrin and metalloproteinase with a thrombospondin type 1 motif, member 13; IGF-1: insulin-like growth factor; IGFBP-3: insulin-like growth factor binding protein 3; NT-proBNP: N-terminal fragment of B-type natriuretic peptide; MRproANP: midregional pro-atrial natriuretic peptide; MMP: matrix metalloproteinase; H- and B-FABP: heart and brain type fatty acid-binding protein; tHy: total homocysteine; NSE: neuron-specific enolase; MBP: myelin-basic protein; BDNF: brain-derived neurotrophic factor; GELS: gelsolin; DRP2: dihydropyrimidinase-related protein-2; CNS: central nervous system; CYTA: cystatin A; NfL: neurofilament light chain; DDK-1: Dicckopf-1

Author	Year	Stroke Type	Serum Biomarker Assessed
Biomarkers of Inflammation				
Christensen et al. [27]	2002	Ischaemic	TNFa IL-1b IL-1RA IL-6 IL-10 TNF-R1 TNF-R2
Smith et al. [28]	2004	Ischaemic	IL-6
Sotgiu et al. [29]	2006	Ischaemic	TNFa ICAM-1 IL-6
Welsh et al. [30]	2009	Ischaemic	IL-6
Smedbakken et al. [31]	2011	Ischaemic	ALCAM
Tsai et al. [32]	2014	Ischaemic	TBARS
Xu et al. [33]	2018	Haemorrhagic	Tim-3
Li et al. [34]	2020	Haemorrhagic	OPN
Acute Phase Reactants				
Di Napoli et al. [35]	2001	Ischaemic	CRP
Christensen et al. [27]	2002	Ischaemic	Ferritin
Montaner et al. [36]	2006	Ischaemic	CRP
den Hertog et al. [37]	2009	Ischaemic	CRP
Welsh et al. [30]	2009	Ischaemic	CRP
Huangfu et al. [38]	2020	Haemorrhagic	SAA
Biomarkers of Haemostasis				
Di Napoli et al. [35]	2001	Ischaemic	Fibrinogen
Nomura et al. [39]	2004	Ischaemic	TM
Jauch et al. [40]	2006	Ischaemic	TM
Carter et al. [41]	2007	Ischaemic	Fibrinogen FVIII beta-TG vWF t-PA FXIII A subunit
Welsh et al. [30]	2009	Ischaemic	d-dimer
Taylor et al. [42]	2020	Ischaemic	vWF:Ag-ADAMTS13Ac ratio
Nuclear Material				
Rainer et al. [43]	2003	All	Plasma DNA
Liang et al. [44]	2019	Ischaemic	microRNA-140-5p
Zuo et al. [45]	2020	Ischaemic	circFUNDC1 circPDS5B circCDC14A
Creatinine				
Carter et al. [41]	2007	Ischaemic	Creatinine
Hormones				
Denti et al. [46]	2004	Ischaemic	IGF-1
Zweifel et al. [47]	2011	Haemorrhagic	GH
Wang et al. [48]	2016	Ischaemic	Copeptin
Armbrust et al. [49]	2017	Ischaemic	IGF-1 IGFBP-3
Yang et al. [50]	2017	Ischaemic	NT-proBNP
De Marchis et al. [51]	2018	Ischaemic	MRproANP
Tu et al. [52]	2018	Ischaemic	Irisin
Arnold et al. [53]	2020	Ischaemic	MRproANP
Amino Acids, Proteins and Enzymes				
Alvarez-Sabin et al. [54]	2004	Haemorrhagic	MMP-3
Wunderlich et al. [55]	2005	Ischaemic	H-FABP B-FABP
Sotgiu et al. [29]	2006	Ischaemic	MMP
Carter et al. [41]	2007	Ischaemic	Albumin Haemoglobin
Yan et al. [56]	2016	Haemorrhagic	Galectin-3
Zhong et al. [57]	2017	Ischaemic	tHy
Zhong et al. [58]	2017	Ischaemic	MMP-9
Zeng et al. [59]	2019	Ischaemic	Galectin-3
Qian et al. [60]	2020	Ischaemic	Endostatin
Zhang et al. [61]	2020	Ischaemic	Endostatin
CNS-Specific Biomarkers				
Abraha et al. [62]	1997	All	S100B
Wunderlich et al. [63]	2006	Ischaemic	NSE Tau protein
Jauch et al. [40]	2006	Ischaemic	S100B NSE MBP
Delgado et al. [64]	2006	Haemorrhagic	S100B
Sotgiu et al. [29]	2006	Ischaemic	BDNF
García-Berrocoso et al. [65]	2013	Ischaemic	GELS DRP2 GELS/DRP2 GELS/DRP2/CYTA
Tiedt et al. [66]	2018	Ischaemic	NfL
Zhu et al. [67]	2019	Ischaemic	DDK-1

Categories of biomarkers studied

Inflammation

As is the case in AIS, the disruption of the supply of oxygen to the brain parenchyma results in focal ischaemia (or necrosis), following which a number of biochemical and cellular changes occur [68]. Necrosis results in the release of reactive oxygen species (ROS) which promotes oxidative stress, and nucleic acids which promote cytokine and chemokine release, which in turn results in the recruitment of microglia [69]. Microglial cells play a critical role in the inflammatory cascade by upregulating the production of a number of proinflammatory chemokines and cytokines, which results in the deleterious effects following the acute insult, perpetuating the cycle [70]. Although fundamentally different in its initial insult, inflammation similarly plays an important role in secondary cellular damage following ICH [71]. These measurable cytokines and chemokines represent a potential quantitative assessment of the extent of the insult, forming the theoretical basis for their use as prognostic biomarkers.

(i) Tumour necrosis factor alpha (TNFα) and its receptors: TNFα, a cytokine produced within the central nervous system (CNS) by both neurones and glial cells, is an activity-dependent cytokine with low levels in the normal physiological state [72]. Often thought of as the prototypical proinflammatory cytokine, it has both advantageous (such as maintaining healthy myelin) and deleterious (such as inducing cellular necrosis) effects, thought to be the result of different cellular receptors [73]. Its usefulness as a prognostic biomarker has yielded contradictory results, which may reflect this pleiotropy [27,29].

(ii) Interleukins (IL): ILs, produced by a myriad of cell types throughout the body, play an imperative role in the homeostasis of inflammatory cellular function, including cellular activation, suppression, proliferation and migration [74]. Functionally, ILs may be considered pro-inflammatory or anti-inflammatory, with levels maintained in a fine balance in normal physiological states. Disruption in this homeostasis results in the deleterious effects seen following cerebral insults [75-77]. Measurement of ILs (and their direct drivers) as tools for prognostication in strokes has resulted in mixed results [27-30,34]. Of all the markers studied, IL-6 appears to have the greatest potential of being clinically useful.

(iii) Adhesion molecules: Adhesion, leukocyte rolling and subsequent cellular transmigration of inflammatory cells are largely regulated by adhesion molecules [78]. Within the CNS, these molecules play a role in neuronal cell migration, synapse formation and inflammation [79]. The production of these molecules is rapidly upregulated following initiation of the inflammatory cascade following cerebral ischaemia [80]. Both activated leukocyte adhesion molecule (ALCAM) and intercellular adhesion molecule 1 (ICAM-1) have shown promising results as biomarkers [29,31].

(iv) T-cell immunoglobulin and mucin-domain: The Tim gene family, expressed on T-cells, B-cells and dendritic cells, underpins the complexity of immune regulation and dysregulation in a number of conditions [81]. The interplay between a number of Tim-subsets results in different immune responses to inflammation and may promote or suppress the inflammatory cascade. Tim-3 has been shown to downregulate the T helper 1 response, thereby acting as an anti-inflammatory molecule [82]. Tim-3 has been studied in ICH and has been found to be potential as both a prognostic biomarker and a potential therapeutic target [33].

(v) Thiobarbituric acid-reactive substances (TBARS): The inflammatory response following cerebral ischaemia is driven, in part, by the production of ROS [83]. Malondialdehyde, a by-product of lipid peroxidation, is produced in excess as part of the inflammatory response following cerebral ischaemia and can be measured, indirectly, by measuring TBARS [84]. Early measurement of TBARS predicts early clinical outcome as well as long-term prognosis following AIS [32].

Acute Phase Reactants (APR)

APR vary greatly in the presence of inflammation and is often a surrogate for the extent of the systemic inflammatory response to various insults [85]. APRs, including C-reactive protein (CRP) and ferritin, are known to increase following cerebral insults [86,87].

(i) C-reactive protein (CRP): CRP, first discovered in 1930, is primarily induced by the presence of IL-6 and produced by the liver [88]. Not only has CRP been found to be increased following stroke, but it has also been shown to be a predictor of new-onset strokes [89]. Results have been fairly conclusive and support its use as a prognostic biomarker following AIS [30,35,36].

(ii) Ferritin: Ferritin is the primary storage form of iron and increases in response to hepcidin [90]. Hepcidin, in turn, is thought to be an APR and has evolved to reduce iron availability, a metabolic rate-limiting step for many pathogens [91]. Ferritin, a commonly analysed laboratory parameter, is therefore thought to serve as a surrogate marker for hepcidin, and therefore inflammation. As such, authors have sought to determine the potential use of ferritin as a prognostic biomarker. Unfortunately, results have not been supportive of this [27]. This may reflect a timing issue in blood sampling due to the delay from the initiation of the inflammatory cascade, increased levels of hepcidin and subsequent increased iron storage as ferritin.

(iii) Serum amyloid A (SAA): Isolated more than half a century ago, SAA has been under much research and has been primarily identified in pathological states, with recent advances showing some role in normal lipid metabolism [92]. Predominantly synthesised in the liver, SAA acts as a pleiotropic immune modulator, with a predominantly pro-inflammatory effect [93]. A recent study by Huangfu et al. revealed the significant predictive value of SAA following ICH [38].

Mediators of Haemostasis

Numerous factors play a critical role in the maintenance of normal, laminar vascular flow to the brain, with disruption in endothelial wall integrity, flow or coagulability resulting in thrombosis [94]. Focal cerebral ischaemia is often due to local hypercoagulability and it can be postulated that the level of activity of mediators of haemostasis represents the extent of thrombosis [95]. Focal coagulopathies appear to play a central role in haematoma formation following ICH, which is directly correlated to functional outcomes [96].

(i) Pro-coagulation: Peripherally measured factors which promote thromboses, such as the von Willebrand factor, factors VIII and XIII and beta-thromboglobulin have been shown to have a significant ability to predict both mortality and morbidity following strokes [41,42]. Studies assessing fibrinogen revealed mixed results [35,41].

(ii) Anti-coagulation: Thrombomodulin, a naturally occurring anticoagulant, found on endothelial cell membranes failed to yield a clinically significant prognostic value [39,40]. Endogenous tissue plasminogen activator activity, however, appeared to predict poor outcomes well [41].

(iii) Coagulation end-products: Following fibrinolysis, d-dimer is produced as a soluble fibrin degradation product and is detectable in the serum [97]. A paper by Welsh et al. provided evidence that d-dimer levels following AIS have a good prognostic value [30].

Nuclear Material

The nuclear material is released following cell death and can be detected using specialised equipment [98]. Primarily utilised in so-called liquid biopsies in cancer detection, free DNA and RNA material have been identified as potential markers for the extent of cerebral damage following strokes, and may therefore predict outcomes. Some studies support this claim when assessing plasma DNA and specific microRNA [43,44]. Research looking at other circular nuclear material has not supported its use as a prognostic biomarker [45].

Creatinine

Creatine, an amino acid-like compound, is predominantly produced in the liver and kidneys, but has been shown to be a key in normal cellular function within the brain [99]. Creatinine, a metabolic by-product of creatine homeostasis, has been widely studied [100,101]. Literature on its use as a prognostic biomarker following strokes is limited, however, it has been shown to be a good predictor of mortality following AIS [41].

Hormones

Various hormones have been shown to have a role in stroke risk as well as the neuroinflammatory response following a stroke [102-104]. This has provided an opportunity to assess a number of hormones as prognostic biomarkers in stroke.

(i) Growth hormone (GH)/insulin-like growth factor-1 (IGF-1) pathway: Released from the anterior pituitary somatotropic cells GH, also known as somatotropin, fulfils a multitude of functions either directly by binding to target cells or indirectly via the action of IGF-1 [105]. Following the initiation of the inflammatory cascade, multiple factors, including GH-responsive genes (such as the rat serine inhibitor 2 locus), suppress the expression of GH and allow for it to be considered a negative APR (meaning levels reduce in the face of inflammation) [106]. Following ICH, however, activation of the hypothalamic-pituitary axis promotes the release of GH, with elevated levels being associated with poor clinical outcomes [47]. Following production in the liver, IGF-1 is bound to IGF-1 binding protein (IGFBP) and acts on IGF-1 receptors to promote cellular growth [107]. An inverse relationship exists between stroke risk and functional outcomes following strokes and IGF-1 and IGFBP-3 levels [108].

(ii) Natriuretic peptides: Natriuretic peptides consist of three structurally similar hormones which fulfil a primary cardioprotective role [109,110]. Elevated levels of both atrial-and brain natriuretic peptides have been associated with stroke risk, aetiology and prognosis [111-113]. A number of studies have confirmed the utility of natriuretic peptides as prognostic biomarkers following strokes [50,51,53].

(iii) Copeptin: Antidiuretic hormone (arginine vasopressin, ADH) is synthesised in the hypothalamus and stored within the posterior pituitary gland where it is released in response to hypovolaemia and hypernatraemia [114]. Measurement of ADH is made difficult due to various technical factors and copeptin, an amino acid glycopeptide, which has shown good potential as a biomarker in various disease states, has been shown to be an accurate surrogate marker for ADH release [115]. Elevated levels of copeptin measured shortly after symptom onset following AIS are a good predictor of both morbidity and mortality [48].

(iv) Irisin: Produced by the enzymatic cleavage of a protein found on myocyte membranes, irisin is a key hormone in the regulation of brown adipocytes [116]. Low levels of irisin are associated with sedentary lifestyles and its relationship with obesity and metabolic regulation has been suggested [117,118]. Reduced irisin levels following AIS are associated with increased psychological morbidity [52].

Amino Acids, Proteins and Enzymes

(i) Homocysteine: The methionine derived amino acid homocysteine is necessary for cellular homeostasis [119]. The association between homocysteine and atherosclerosis dates back to the late 1960s with stroke complicating hyperhomocysteinaemia [120]. In addition to its role in the pathogenesis of the cardiovascular disease, homocysteine levels have been found useful in predicting poor clinical outcomes following AIS [57].

(ii) Haemoglobin: The iron-containing protein haemoglobin is essential for intravascular oxygen transport and delivery [121,122]. Aberrant haemoglobin concentrations, both abnormally high and low, are associated with strokes in all stages of its pathophysiology [123-126]. Low haemoglobin levels are predictive of mortality following cerebral ischaemia [41].

(iii) Albumin: As the most abundant plasma protein, albumin fulfils a number of functions [127]. Acting as a negative APR, albumin has long been considered a useful tool in determining the extent of inflammation [85]. Carter et al. found that albumin is a good prognostic marker of mortality following AIS [41]. Hypoalbuminaemia may represent a poor pre-stroke physiological reserve, which increases the risk of death following a cerebral insult [128].

(iv) Fatty acid-binding protein (FABP): To date, nine FABPs have been identified and form part of the intracellular lipid-binding protein family and are involved in the binding and trafficking of intracellular hydrophobic ligands [129,130]. The heart-type FABP is predominantly found within cardiac myocytes and has been shown to be a good prognostic biomarker following both myocardial ischaemia as well as AIS, whilst the brain-type FABP has been shown to provide significant prognostic value following AIS [55,131].

(v) Galectin: The family of beta-galactoside-binding animal lectins, galectin, has been shown to be involved in a number of physiological and disease processes [132]. Some classes of galectins are predominantly expressed within the brain and are fundamental in the formation and migration of specific neuronal tissue following injury [133]. Galectin-3, a pleiotropic molecule, has in recent years been the target of a number of investigational therapeutics in a wide variety of conditions [134-136]. Its use as a potential diagnostic and prognostic biomarker in cardiovascular disease, especially, has been proven [56,59,137].

(vi) Matrix metalloproteinase (MMP): The zinc-dependent family of enzymes, MMPs, are critical in maintaining allostasis within the extracellular matrix [138]. Of the more than 20 MMPs, a number of them have become important biomarkers in a host of diseases and may prove a therapeutic target in future, including strokes [139,140]. MMP polymorphisms have been identified as an important consideration in both the pathophysiological processes and clinical outcomes following strokes [141]. MMP-9 in particular has been shown to be a key factor in the disruption of the blood-brain barrier following strokes and is associated with stroke severity [142]. Its role in prognostication following both ICH and AIS has been proven [29,54].

(vii) Endostatin: The angiogenic response following cerebral ischaemia appears to be an important defensive reaction and has a direct effect on long-term neurological recovery [143]. The mechanisms underpinning angiogenesis are complex and rely on the balance of stimulating and inhibitory factors [144]. Endostatin, a potent inhibitor of angiogenesis found in vascular walls and basement membranes, is associated with poor functional outcomes as well as mortality following AIS [60,61].

CNS-Specific Biomarkers

(i) S100B: Concentrated within glial cells, S100B functions as a calcium-binding protein with a not yet fully understood role [145]. Its clinical use as a marker of neurological disorders has been well established, with elevated levels both in the CSF and in the peripheral circulation representing active disease or neuronal damage [146,147]. S100B reliably predicts the severity of the neuronal injury, however, its utility as a prognostic biomarker in stroke has yielded mixed results [40,62,64,148].

(ii) Neuron-specific enolase (NSE): One of three isoenzymes of enolase, NSE serves a critical role in neuronal differentiation [149]. NSE has found significant utility in a number of neurological and oncological disorders [150,151]. Its clinical utility in stroke-related cerebral insults has resulted in mixed evidence from literature [40,63].

(iii) Wnt pathway: The Wnt signalling pathway is crucial to cellular regulation, including cell migration, cell polarity and neural patterning [152]. The increased expression of the Wnt antagonist, Dicckopf-1 (DDK-1), results from neurodegenerative processes and in further neurodegeneration via a complex pathway resulting in cellular death (due to the inhibition of BCL-2 expression and induction of BAX) [153]. This canonical loss of Wnt signalling results in the phosphorylation of tau protein [154]. The microtubule-associated neuronal protein, tau, is phosphorylated under normal physiological conditions, however, excessive phosphorylation results in self-aggregation resulting in tauopathies [155]. The build-up of these oligomers is associated with increased morbidity and mortality and supports the rationale for the use of both DDK-1 and tau as prognostic biomarkers [63,67].

(iv) Neurofilament light chain (NfL): Neurofilaments are particularly abundant in axons, which are dependent on NfL to maintain axonal diameter, and are detectable in pathological states due to neuronal cell death [156,157]. The high translational value of NfL has promoted its use as a prognostic biomarker in a host of neurodegenerative disorders, including multiple sclerosis [158-161]. Its utility as a prognostic biomarker in stroke has been confirmed by Tiedt et al. [66].

(v) Brain-derived neurotrophic factor (BDNF): Neuronal plasticity requires BDNF, a highly regulated molecule, which shows great variability in both health and disease [162]. It is predominantly expressed in the CNS and the gut and is involved in regulating energy metabolism and upregulating pro-survival genes, with reduced levels associated with neurodegenerative disorders [163]. The relationship between BDNF and functional outcomes following AIS has been shown [29].

(vi) Myelin-basic protein (MBP): Myelin formation within the CNS is highly dependent on the activity of MBP, and oligodendrocyte differentiation requires fine regulation of MBP expression [164]. MBP readily interacts with a host of other proteins allowing it to participate in transmission of extracellular signals [165]. Elevated central and peripheral levels of MBP are associated with cerebral damage [166]. MBP does not increase early following the onset of cerebral ischaemia and this may explain why it has not been found to be a useful biomarker in stroke [40,167].

(vii) Novel brain-derived biomarkers: Gelsolin, dihydropyrimidinase-related protein-2 and cystatin A have been identified by researchers following experimental identification in animal studies, with human post-mortem confirmation [65]. Although the function of these molecules is yet to be determined, the authors found that their presence following a stroke is associated with poor functional outcomes.

Applications in South Africa

Successful management of stroke, including diagnosis and treatment decision-making, is highly time-sensitive [168-170]. Neuroradiological services are not readily accessible to many communities in SA, notably communities that rely on public healthcare, and significant delays in accessing facilities that provide these services are experienced. This affects time-to-diagnosis and time-to-treatment which, specifically in stroke medicine, may result in a significant delay in patient care, and poorer outcomes. Biomarkers may provide an additive tool; however, limitations remain. Although some readily available biochemical tests have shown significant potential in predicting patient outcomes following stroke, cost and availability limit their use. For instance, the GH and NT-proBNP tests cost ZAR121.16 (approximately US$8) and ZAR509.45 (approximately US$32), respectively [171].

Some biomarkers, including SAA and IGF-1 are available, however, are not being widely utilised. These tests require specialised equipment, which is often only available at specialist centres. The cost analysis, therefore, needs to include the transport of the samples in addition to the laboratory cost, which, as in the case of IGF-1, can be nearly ZAR500 (approximately US$32) [171]. Potential biomarkers that have been identified in diseases other than strokes, such as copeptin, DDK-1 and MRproANP, were reported in multiple articles suggesting wider applicability in strokes. These tests are not readily, if at all, available in the private and public healthcare sectors in SA. The difficulty and cost of these tests make it unlikely that they will be available anytime soon [172]. The significant disparity between LMIC and high-income countries poses a barrier to their effective implementation of novel biomarkers in the near future [173].

The delay from blood sampling to the availability of a biomarker result must also be considered. The currently available biomarkers in Uganda (an LMIC), GH and IGF, for instance, have a turnaround time of 2-3 days and 3-5 days, respectively [174]. Although its diagnostic potential is severely impacted by this delay, it might still be of prognostic value.

People within SA can access either public or private healthcare, with the former providing care from a resource-limited setting. The introduction of the National Health Insurance bill will likely result in the private healthcare sector sharing the financial burden, and the role of cost-effective adjuncts in managing patients with stroke will become ever more important.

Implications and recommendations

Cerebrovascular events, whether ischaemic or haemorrhagic in nature, result in significant morbidity and mortality worldwide [175]. Early detection and risk stratification yield improved patient outcomes [6,7]. Numerous clinical and radiological scoring systems have been introduced to determine the outcome for patients following a stroke; however, the availability of neuroradiological imaging in LMIC greatly limits its implementation. McLane et al. compared the availability of neurodiagnostic tools, such as MRI and CT, between LMIC and wealthier nations and revealed a significant disparity, “diagnostic gap” [176]. Novel approaches are required to bridge this gap.

The availability of objective, measurable biomarkers can provide accurate prognostication and identify high-risk individuals following a stroke. This is especially true in communities where access to specialised testing is limited. The advent of point-of-care testing, for markers such as creatine kinase (CK) and amino-terminal pro-peptide counterpart (NT-proBNP), has been shown to have a significantly positive impact on health outcomes in resource-limited settings and should be further researched [9,177].

## Conclusions

This scoping review revealed the currently available biomarkers from published literature. Laboratory investigations range from widely utilised tests to newly identified experimental tests. The applicability in SA, based on cost and availability, varies greatly. The scoping review has limitations. First, publication bias needs to be considered. Research that has either not been submitted due to statistically insignificant results or research declined by journals cannot be assessed. Second, the reviewers are not fluent in languages other than English, limiting the review of articles published in languages such as Mandarin, Spanish, French, German or Japanese.

Biomarkers show tremendous promise in aiding clinicians in the early prognostication of patients following cerebrovascular events. This scoping review highlights the need for further research to be performed to assess new biomarkers, in terms of both clinical and laboratory accuracy and cost-effectiveness, which are readily available in the SA setting.

## References

[1] Virani SS, Alonso A, Benjamin EJ (2020). Heart disease and stroke statistics—2020 update: a report from the American Heart Association. Circulation.

[2] Yan LL, Li C, Chen J (2016). Prevention, management, and rehabilitation of stroke in low- and middle-income countries. eNeurologicalSci.

[3] (2021). World Bank Country and Lending Groups. Online.

[4] Maredza M, Bertram MY, Tollman SM (2015). Disease burden of stroke in rural South Africa: an estimate of incidence, mortality and disability adjusted life years. BMC Neurol.

[5] Maredza M, Chola L (2016). Economic burden of stroke in a rural South African setting. eNeurologicalSci.

[6] Phipps MS, Cronin CA (2020). Management of acute ischemic stroke. BMJ.

[7] Marler JR, Tilley BC, Lu M (2000). Early stroke treatment associated with better outcome: the NINDS rt-PA stroke study. Neurology.

[8] Luppa PB, Müller C, Schlichtiger A, Schlebusch H (2011). Point-of-care testing (POCT): Current techniques and future perspectives. Trends Analyt Chem.

[9] Bustamante A, García-Berrocoso T, Rodriguez N, Llombart V, Ribó M, Molina C, Montaner J (2016). Ischemic stroke outcome: a review of the influence of post-stroke complications within the different scenarios of stroke care. Eur J Intern Med.

[10] Strimbu K, Tavel JA (2010). What are biomarkers?. Curr Opin HIV AIDS.

[11] Puntmann VO (2009). How-to guide on biomarkers: biomarker definitions, validation and applications with examples from cardiovascular disease. Postgrad Med J.

[12] FDA-NIH Biomarker Working Group (2016). BEST (Biomarkers, EndpointS, and other Tools) Resource. Silver Spring.

[13] Misra S, Montaner J, Ramiro L (2020). Blood biomarkers for the diagnosis and differentiation of stroke: a systematic review and meta-analysis. Int J Stroke.

[14] Misra S, Kumar A, Kumar P (2017). Blood-based protein biomarkers for stroke differentiation: a systematic review. Proteomics Clin Appl.

[15] Whiteley W, Tseng MC, Sandercock P (2008). Blood biomarkers in the diagnosis of ischemic stroke: a systematic review. Stroke.

[16] Martin AJ, Price CI (2018). A systematic review and meta-analysis of molecular biomarkers associated with early neurological deterioration following acute stroke. Cerebrovasc Dis.

[17] Dolmans LS, Rutten FH, Koenen NC, Bartelink ME, Reitsma JB, Kappelle LJ, Hoes AW (2019). Candidate biomarkers for the diagnosis of transient ischemic attack: a systematic review. Cerebrovasc Dis.

[18] Donkel SJ, Benaddi B, Dippel DW, Ten Cate H, de Maat MP (2019). Prognostic hemostasis biomarkers in acute ischemic stroke. Arterioscler Thromb Vasc Biol.

[19] Jickling GC, Sharp FR (2015). Biomarker panels in ischemic stroke. Stroke.

[20] Levac D, Colquhoun H, O'Brien KK (2010). Scoping studies: advancing the methodology. Implement Sci.

[21] Peterson J, Pearce PF, Ferguson LA, Langford CA (2017). Understanding scoping reviews: definition, purpose, and process. J Am Assoc Nurse Pract.

[22] Arksey H, O'Malley L (2005). Scoping studies: towards a methodological framework. Int J Soc Res Methodol.

[23] Pham MT, Rajić A, Greig JD, Sargeant JM, Papadopoulos A, McEwen SA (2014). A scoping review of scoping reviews: advancing the approach and enhancing the consistency. Res Synth Methods.

[24] Mays N, Roberts E, Popay J (2001). Synthesising Research Evidence. Studying the organisation and delivery of health services: research methods.

[25] Moher D, Liberati A, Tetzlaff J, Altman DG, The PRISMA Group (2009). Preferred reporting items for systematic reviews and meta-analyses: the PRISMA statement. PLoS Med.

[26] Young JM, Solomon MJ (2009). How to critically appraise an article. Nat Clin Pract Gastroenterol Hepatol.

[27] Christensen H, Boysen G, Johannesen HH, Christensen E, Bendtzen K (2002). Deteriorating ischaemic stroke. cytokines, soluble cytokine receptors, ferritin, systemic blood pressure, body temperature, blood glucose, diabetes, stroke severity, and CT infarction-volume as predictors of deteriorating ischaemic stroke. J Neurol Sci.

[28] Smith CJ, Emsley HC, Gavin CM (2004). Peak plasma interleukin-6 and other peripheral markers of inflammation in the first week of ischaemic stroke correlate with brain infarct volume, stroke severity and long-term outcome. BMC Neurol.

[29] Sotgiu S, Zanda B, Marchetti B (2006). Inflammatory biomarkers in blood of patients with acute brain ischemia. Eur J Neurol.

[30] Welsh P, Barber M, Langhorne P, Rumley A, Lowe GD, Stott DJ (2009). Associations of inflammatory and haemostatic biomarkers with poor outcome in acute ischaemic stroke. Cerebrovasc Dis.

[31] Smedbakken L, Jensen JK, Hallén J (2011). Activated leukocyte cell adhesion molecule and prognosis in acute ischemic stroke. Stroke.

[32] Tsai NW, Chang YT, Huang CR (2014). Association between oxidative stress and outcome in different subtypes of acute ischemic stroke. Biomed Res Int.

[33] Xu C, Ge H, Wang T, Qin J, Liu Liu, Liu Y (2018). Increased expression of T cell immunoglobulin and mucin domain 3 on CD14+ monocytes is associated with systemic inflammatory reaction and brain injury in patients with spontaneous intracerebral hemorrhage. J Stroke Cerebrovasc Dis.

[34] Li HJ, Han NN, Nan Y, Zhang K, Li G, Chen H (2020). Plasma osteopontin acts as a prognostic marker in acute intracerebral hemorrhage patients. Clin Chim Acta.

[35] Di Napoli M, Papa F, Bocola V (2001). Prognostic influence of increased C-reactive protein and fibrinogen levels in ischemic stroke. Stroke.

[36] Montaner J, Fernandez-Cadenas I, Molina CA (2006). Poststroke C-reactive protein is a powerful prognostic tool among candidates for thrombolysis. Stroke.

[37] den Hertog HM, van Rossum JA, van der Worp HB (2009). C-reactive protein in the very early phase of acute ischemic stroke: association with poor outcome and death. J Neurol.

[38] Huangfu XQ, Wang LG, Le ZD, Tao B (2020). Utility of serum amyloid A as a potential prognostic biomarker of acute primary basal ganglia hemorrhage. Clin Chim Acta.

[39] Nomura E, Kohriyama T, Kozuka K, Kajikawa H, Nakamura S, Matsumoto M (2004). Significance of serum soluble thrombomodulin level in acute cerebral infarction. Eur J Neurol.

[40] Jauch EC, Lindsell C, Broderick J, Fagan SC, Tilley BC, Levine SR, for the NINDS rt-PA Stroke Study Group (2006). Association of serial biochemical markers with acute ischemic stroke: the National Institute of Neurological Disorders and Stroke recombinant tissue plasminogen activator stroke study. Stroke.

[41] Carter AM, Catto AJ, Mansfield MW, Bamford JM, Grant PJ (2007). Predictive variables for mortality after acute ischemic stroke. Stroke.

[42] Taylor A, Vendramin C, Singh D, Brown MM, Scully M (2020). von Willebrand factor/ADAMTS13 ratio at presentation of acute ischemic brain injury is predictive of outcome. Blood Adv.

[43] Rainer TH, Wong LKS, Lam W, Yuen E, Lam NYL, Metreweli C, Lo YMD (2003). Prognostic use of circulating plasma nucleic acid concentrations in patients with acute stroke. Clin Chem.

[44] Liang HB, He JR, Tu XQ, Ding KQ, Yang GY, Zhang Y, Zeng LL (2019). MicroRNA- 140-5p: a novel circulating biomarker for early warning of late-onset post-stroke depression. J Psychiatr Res.

[45] Zuo L, Zhang L, Zu J (2020). Circulating circular RNAs as biomarkers for the diagnosis and prediction of outcomes in acute ischemic stroke. Stroke.

[46] Denti L, Annoni V, Cattadori E (2004). Insulin-like growth factor 1 as a predictor of ischemic stroke outcome in the elderly. Am J Med.

[47] Zweifel C, Katan M, Schuetz P, Ernst A, Mariani L, Müller B, Christ-Crain M (2011). Growth hormone and outcome in patients with intracerebral hemorrhage: a pilot study. Biomarkers.

[48] Wang CB, Zong M, Lu SQ, Tian Z (2016). Plasma copeptin and functional outcome in patients with ischemic stroke and type 2 diabetes. J Diabetes Complicat.

[49] Armbrust M, Worthmann H, Dengler R (2017). Circulating insulin-like growth factor-1 and insulin-like growth factor binding protein-3 predict three-months outcome after ischemic stroke. Exp Clin Endocrinol Diabetes.

[50] Yang J, Zhong C, Wang A (2017). Association between increased N-terminal pro-brain natriuretic peptide level and poor clinical outcomes after acute ischemic stroke. J Neurol Sci.

[51] De Marchis GM, Schneider J, Weck A (2018). Midregional proatrial natriuretic peptide improves risk stratification after ischemic stroke. Neurology.

[52] Tu WJ, Qiu HC, Liu Q, Li X, Zhao JZ, Zeng X (2018). Decreased level of irisin, a skeletal muscle cell-derived myokine, is associated with post-stroke depression in the ischemic stroke population. J Neuroinflammation.

[53] Arnold M, Nakas C, Luft A, Christ-Crain M, Leichtle A, Katan M (2020). Independent prognostic value of MRproANP (Midregional Proatrial Natriuretic Peptide) levels in patients with stroke is unaltered over time. Stroke.

[54] Alvarez-Sabin J, Delgado P, Abilleira S (2004). Temporal profile of matrix metalloproteinases and their inhibitors after spontaneous intracerebral hemorrhage: relationship to clinical and radiological outcome. Stroke.

[55] Wunderlich MT, Hanhoff T, Goertler M, Spener F, Glatz JF, Wallesch CW, Pelsers MM (2005). Release of brain-type and heart-type fatty acid-binding proteins in serum after acute ischaemic stroke. J Neurol.

[56] Yan XJ, Yu GF, Jie YQ, Fan XF, Huang Q, Dai WM (2016). Role of galectin-3 in plasma as a predictive biomarker of outcome after acute intracerebral hemorrhage. J Neurol Sci.

[57] Zhong C, Xu T, Xu T (2017). Plasma homocysteine and prognosis of acute ischemic stroke: a gender-specific analysis from CATIS randomized clinical trial. Mol Neurobiol.

[58] Zhong C, Yang J, Xu T (2017). Serum matrix metalloproteinase-9 levels and prognosis of acute ischemic stroke. Neurology.

[59] Zeng N, Wang A, Xu T (2019). Co-effect of serum galectin-3 and high-density lipoprotein cholesterol on the prognosis of acute ischemic stroke. J Stroke Cerebrovasc Dis.

[60] Qian S, Li R, Zhang C (2020). Plasma endostatin levels at acute phase of ischemic stroke are associated with post-stroke cognitive impairment. Neurotox Res.

[61] Zhang C, Qian S, Zhang R (2020). Endostatin as a novel prognostic biomarker in acute ischemic stroke. Atherosclerosis.

[62] Abraha HD, Butterworth RJ, Bath PM, Wassif WS, Garthwaite J, Sherwood RA (1997). Serum S-100 protein, relationship to clinical outcome in acute stroke. Ann Clin Biochem.

[63] Wunderlich MT, Lins H, Skalej M, Wallesch CW, Goertler M (2006). Neuron-specific enolase and tau protein as neurobiochemical markers of neuronal damage are related to early clinical course and long-term outcome in acute ischemic stroke. Clin Neurol Neurosurg.

[64] Delgado P, Sabin JA, Santamarina E, Molina CA, Quintana M, Rosell A, Montaner J (2006). Plasma S100B level after acute spontaneous intracerebral hemorrhage. Stroke.

[65] García-Berrocoso T, Penalba A, Boada C (2013). From brain to blood: Nenw biomarkers for ischemic stroke prognosis. J Proteomics.

[66] Tiedt S, Duering M, Barro C (2018). Serum neurofilament light: a biomarker of neuroaxonal injury after ischemic stroke. Neurology.

[67] Zhu Z, Guo D, Zhong C (2019). Serum Dkk-1 (Dickkopf-1) is a potential biomarker in the prediction of clinical outcomes among patients with acute ischemic stroke. Arterioscler Thromb Vasc Biol.

[68] Dietrich WD (2017). Chapter 22 - Histopathology of Cerebral Ischemia and Stroke. Primer on Cerebrovascular Diseases (Second Edition).

[69] Kawabori M, Yenari MA (2015). Inflammatory responses in brain ischemia. Curr Med Chem.

[70] Qin C, Zhou LQ, Ma XT (2019). Dual functions of microglia in ischemic stroke. Neurosci Bull.

[71] Wang J, Doré S (2007). Inflammation after intracerebral hemorrhage. J Cereb Blood Flow Metab.

[72] Pan W, Zadina JE, Harlan RE, Weber JT, Banks WA, Kastin AJ (1997). Tumor necrosis factor-alpha: a neuromodulator in the CNS. Neurosci Biobehav Rev.

[73] Probert L (2015). TNF and its receptors in the CNS: the essential, the desirable and the deleterious effects. Neuroscience.

[74] Justiz Vaillant J, Qurie A (2021). Interleukin. StatPearls. Treasure Island.

[75] Murray KN, Parry-Jones AR, Allan SM (2015). Interleukin-1 and acute brain injury. Front Cell Neurosci.

[76] Stein DM, Lindel AL, Murdock KR, Kufera JA, Menaker J, Scalea TM (2012). Use of serum biomarkers to predict secondary insults following severe traumatic brain injury. Shock.

[77] Betz AL, Schielke GP, Yang GY (1996). Interleukin-1 in cerebral ischemia. Keio J Med.

[78] Ren G, Roberts AI, Shi Y (2011). Adhesion molecules: key players in Mesenchymal stem cell-mediated immunosuppression. Cell Adh Migr.

[79] Togashi H, Sakisaka T, Takai Y (2009). Cell adhesion molecules in the central nervous system. Cell Adh Migr.

[80] Yilmaz G, Granger DN (2008). Cell adhesion molecules and ischemic stroke. Neurol Res.

[81] Li Z, Ju Z, Frieri M (2013). The T-cell immunoglobulin and mucin domain (Tim) gene family in asthma, allergy, and autoimmunity. Allergy Asthma Proc.

[82] Zhang XM, Shan NN (2014). The role of T cell immunoglobulin and mucin domain-3 in immune thrombocytopenia. Scand J Immunol.

[83] Samson Y, Lapergue B, Hosseini H (2005). Inflammation and ischaemic stroke: current status and future perspectives. (Article in French). Rev Neurol.

[84] Catalán V, Frühbeck G, Gómez-Ambrosi J (2018). Chapter 8 - Inflammatory and Oxidative Stress Markers in Skeletal Muscle of Obese Subjects. https://doi.org/10.1016/B978-0-12-812504-5.00008-8.

[85] Gulhar R, Ashraf MA, Jialal I (2021). Physiology, Acute Phase Reactants. StatPearls. Treasure Island.

[86] Tamam Y, Iltumur K, Apak I (2005). Assessment of acute phase proteins in acute ischemic stroke. Tohoku J Exp Med.

[87] Young AB, Ott LG, Beard D, Dempsey RJ, Tibbs PA, McClain CJ (1988). The acute-phase response of the brain-injured patient. J Neurosurg.

[88] Nehring SM, Goyal A, Patel BC (2022). C Reactive Protein. StatPearls.

[89] Di Napoli M, Papa F, Bocola V (2001). C-reactive protein in ischemic stroke: an independent prognostic factor. Stroke.

[90] Knovich MA, Storey JA, Coffman LG, Torti SV, Torti FM (2009). Ferritin for the clinician. Blood Rev.

[91] Taketani S (2005). Aquisition, mobilization and utilization of cellular iron and heme: endless findings and growing evidence of tight regulation. Tohoku J Exp Med.

[92] Sack GH Jr (2018). Serum amyloid A - a review. Mol Med.

[93] Eklund KK, Niemi K, Kovanen PT (2012). Immune functions of serum amyloid A. Crit Rev Immunol.

[94] Kushner A, West WP, Pillarisetty LS (2022). Virchow Triad. StatPearls.

[95] Sfredel MD, Burada E, Cătălin B, Dinescu V, Târtea G, Iancău M, Osiac E (2018). Blood coagulation following an acute ischemic stroke. Curr Health Sci J.

[96] Emiru T, Bershad EM, Zantek ND, Datta YH, Rao GH, Hartley EW, Divani AA (2013). Intracerebral hemorrhage: a review of coagulation function. Clin Appl Thromb Hemost.

[97] Johnson ED, Schell JC, Rodgers GM (2019). The D-dimer assay. Am J Hematol.

[98] Drag MH, Kilpeläinen TO (2021). Cell-free DNA and RNA—measurement and applications in clinical diagnostics with focus on metabolic disorders. Physiol Genomics.

[99] Andres RH, Ducray AD, Schlattner U, Wallimann T, Widmer HR (2008). Functions and effects of creatine in the central nervous system. Brain Res Bull.

[100] Delanaye P, Cavalier E, Pottel H (2017). Serum creatinine: not so simple!. Nephron.

[101] Kashani K, Rosner MH, Ostermann M (2020). Creatinine: from physiology to clinical application. Eur J Intern Med.

[102] Perez-Alvarez MJ, Wandosell F (2016). Stroke and neuroinflamation: role of sexual hormones. Curr Pharm Des.

[103] O'Neill PA, Davies I, Fullerton KJ, Bennett D (1991). Stress hormone and blood glucose response following acute stroke in the elderly. Stroke.

[104] Lillicrap T, Garcia-Esperon C, Walker FR (2018). Growth hormone deficiency is frequent after recent stroke. Front Neurol.

[105] Brinkman JE, Tariq MA, Leavitt L, Sharma S (2021). Physiology, Growth Hormone. https://www.ncbi.nlm.nih.gov/books/NBK482141/.

[106] Bergad PL, Schwarzenberg SJ, Humbert JT, Morrison M, Amarasinghe S, Towle HC, Berry SA (2000). Inhibition of growth hormone action in models of inflammation. Am J Physiol Cell Physiol.

[107] Laron Z (2001). Insulin-like growth factor 1 (IGF-1): a growth hormone. Mol Pathol.

[108] Kooijman R, Sarre S, Michotte Y, De Keyser J (2009). Insulin-like growth factor I: a potential neuroprotective compound for the treatment of acute ischemic stroke?. Stroke.

[109] Potter LR, Yoder AR, Flora DR, Antos LK, Dickey DM (2009). Natriuretic Peptides: Their Structures, Receptors, Physiologic Functions and Therapeutic Applications. cGMP: Generators, Effectors and Therapeutic Implications. Handbook of Experimental Pharmacology.

[110] Nishikimi T, Maeda N, Matsuoka H (2006). The role of natriuretic peptides in cardioprotection. Cardiovasc Res.

[111] Cushman M, Judd SE, Howard VJ (2014). N-terminal pro-B-type natriuretic peptide and stroke risk: the reasons for geographic and racial differences in stroke cohort. Stroke.

[112] Vesely DL (2001). Atrial natriuretic peptides in pathophysiological diseases. Cardiovasc Res.

[113] Wang TJ, Larson MG, Levy D (2004). Plasma natriuretic peptide levels and the risk of cardiovascular events and death. N Engl J Med.

[114] Cuzzo B, Padala SA, Lappin SL (2021). Physiology, Vasopressin. StatPearls.

[115] Morgenthaler NG, Struck J, Jochberger S, Dünser MW (2008). Copeptin: clinical use of a new biomarker. Trends Endocrinol Metab.

[116] Korta P, Pocheć E, Mazur-Biały A (2019). Irisin as a multifunctional protein: implications for health and certain diseases. Medicina.

[117] Mai S, Grugni G, Mele C (2020). Irisin levels in genetic and essential obesity: clues for a potential dual role. Sci Rep.

[118] Chen N, Li Q, Liu J, Jia S (2016). Irisin, an exercise-induced myokine as a metabolic regulator: an updated narrative review. Diabetes Metab Res Rev.

[119] Fowler B (2005). Homocysteine: overview of biochemistry, molecular biology, and role in disease processes. Semin Vasc Med.

[120] Kumar A, Palfrey HA, Pathak R, Kadowitz PJ, Gettys TW, Murthy SN (2017). The metabolism and significance of homocysteine in nutrition and health. Nutr Metab.

[121] Gell DA (2018). Structure and function of haemoglobins. Blood Cells Mol Dis.

[122] Ahmed MH, Ghatge MS, Safo MK (2020). Hemoglobin: structure, function and allostery. Subcell Biochem.

[123] Panwar B, Judd SE, Warnock DG, McClellan WM, Booth JN 3rd, Muntner P, Gutiérrez OM (2016). Hemoglobin concentration and risk of incident stroke in community-living adults. Stroke.

[124] Al-Harbi N, Alrasheedi MS, Alshammari ST (2020). Hemoglobin level is associated with severe stroke among stroke patients in Saudi Arabia. Int J Health Sci (Qassim).

[125] Barlas RS, Honney K, Loke YK (2016). Impact of hemoglobin levels and anemia on mortality in acute stroke: analysis of UK Regional Registry Data, systematic review, and meta-analysis. J Am Heart Assoc.

[126] Yotsueda R, Tanaka S, Taniguchi M (2018). Hemoglobin concentration and the risk of hemorrhagic and ischemic stroke in patients undergoing hemodialysis: the Q-cohort study. Nephrol Dial Transplant.

[127] Moman RN, Gupta N, Varacallo M (2021). Physiology, Albumin. StatPearls.

[128] Gounden V, Vashisht R, Jialal I (2021). Hypoalbuminemia. StatPearls. Treasure Island.

[129] Smathers RL, Petersen DR (2011). The human fatty acid-binding protein family: evolutionary divergences and functions. Hum Genomics.

[130] Chmurzyńska A (2006). The multigene family of fatty acid-binding proteins (FABPs): function, structure and polymorphism. J Appl Genet.

[131] Ye XD, He Y, Wang S, Wong GT, Irwin MG, Xia Z (2018). Heart-type fatty acid binding protein (H-FABP) as a biomarker for acute myocardial injury and long-term post-ischemic prognosis. Acta Pharmacol Sin.

[132] Yang RY, Rabinovich GA, Liu FT (2008). Galectins: structure, function and therapeutic potential. Expert Rev Mol Med.

[133] Nio-Kobayashi J, Itabashi T (2021). Galectins and their ligand glycoconjugates in the central nervous system under physiological and pathological conditions. Front Neuroanat.

[134] Krześlak A, Lipińska A (2004). Galectin-3 as a multifunctional protein. Cell Mol Biol Lett.

[135] Caniglia JL, Guda MR, Asuthkar S, Tsung AJ, Velpula KK (2020). A potential role for Galectin-3 inhibitors in the treatment of COVID-19. PeerJ.

[136] Slack RJ, Mills R, Mackinnon AC (2021). The therapeutic potential of galectin-3 inhibition in fibrotic disease. Int J Biochem Cell Biol.

[137] Venkatraman A, Hardas S, Patel N, Singh Bajaj N, Arora G, Arora P (2018). Galectin-3: an emerging biomarker in stroke and cerebrovascular diseases. Eur J Neurol.

[138] Malemud CJ (2006). Matrix metalloproteinases (MMPs) in health and disease: an overview. Front Biosci.

[139] Cabral-Pacheco GA, Garza-Veloz I, Castruita-De la Rosa C (2020). The roles of matrix metalloproteinases and their inhibitors in human diseases. Int J Mol Sci.

[140] Yang Y, Rosenberg GA (2015). Matrix metalloproteinases as therapeutic targets for stroke. Brain Res.

[141] Chang JJ, Stanfill A, Pourmotabbed T (2016). The role of matrix metalloproteinase polymorphisms in ischemic stroke. Int J Mol Sci.

[142] Turner RJ, Sharp FR (2016). Implications of MMP9 for blood brain barrier disruption and hemorrhagic transformation following ischemic stroke. Front Cell Neurosci.

[143] Zhu H, Zhang Y, Zhong Y, Ye Y, Hu X, Gu L, Xiong X (2021). Inflammation-mediated angiogenesis in ischemic stroke. Front Cell Neurosci.

[144] Karamysheva AF (2008). Mechanisms of angiogenesis. Biochemistry (Moscow).

[145] Michetti F, Corvino V, Geloso MC, Lattanzi W, Bernardini C, Serpero L, Gazzolo D (2012). The S100B protein in biological fluids: more than a lifelong biomarker of brain distress. J Neurochem.

[146] Yardan T, Erenler AK, Baydin A, Aydin K, Cokluk C (2011). Usefulness of S100B protein in neurological disorders. J Pak Med Assoc.

[147] Astrand R, Undén J (2019). Clinical use of the calcium-binding S100B protein, a biomarker for head injury. Methods Mol Biol.

[148] Bloomfield SM, McKinney J, Smith L, Brisman J (2007). Reliability of S100B in predicting severity of central nervous system injury. Neurocrit Care.

[149] Isgrò MA, Bottoni P, Scatena R (2015). Neuron-specific enolase as a biomarker: biochemical and clinical aspects. Adv Exp Med Biol.

[150] van Veenendaal LM, Bertolli E, Korse CM, Klop WM, Tesselaar ME, van Akkooi AC (2021). The clinical utility of neuron-specific enolase (NSE) serum levels as a biomarker for Merkel cell carcinoma (MCC). Ann Surg Oncol.

[151] Shaik AJ, Reddy K, Mohammed N, Tandra SR, Rukmini Mridula K, Baba KSS (2019). Neuron specific enolase as a marker of seizure related neuronal injury. Neurochem Int.

[152] Komiya Y, Habas R (2008). Wnt signal transduction pathways. Organogenesis.

[153] Scali C, Caraci F, Gianfriddo M (2006). Inhibition of Wnt signaling, modulation of Tau phosphorylation and induction of neuronal cell death by DKK1. Neurobiol Dis.

[154] Tapia-Rojas C, Inestrosa NC (2018). Loss of canonical Wnt signaling is involved in the pathogenesis of Alzheimer's disease. Neural Regen Res.

[155] Guo T, Noble W, Hanger DP (2017). Roles of tau protein in health and disease. Acta Neuropathol.

[156] Lobsiger CS, Cleveland DW (2009). Neurofilaments: Organization and Function in Neurons. Encyclopedia of Neuroscience. Encyclopedia of Neuroscience.

[157] Yuan A, Rao MV, Veeranna Veeranna, Nixon RA (2012). Neurofilaments at a glance. J Cell Sci.

[158] Loeffler T, Schilcher I, Flunkert S, Hutter-Paier B (2020). Neurofilament-light chain as biomarker of neurodegenerative and rare diseases with high translational value. Front Neurosci.

[159] Zucchi E, Bonetto V, Sorarù G, Martinelli I, Parchi P, Liguori R, Mandrioli J (2020). Neurofilaments in motor neuron disorders: towards promising diagnostic and prognostic biomarkers. Mol Neurodegener.

[160] Gaetani L, Blennow K, Calabresi P, Di Filippo M, Parnetti L, Zetterberg H (2019). Neurofilament light chain as a biomarker in neurological disorders. J Neurol Neurosurg Psychiatry.

[161] Verde F, Steinacker P, Weishaupt JH (2019). Neurofilament light chain in serum for the diagnosis of amyotrophic lateral sclerosis. J Neurol Neurosurg Psychiatry.

[162] Miranda M, Morici JF, Zanoni MB, Bekinschtein P (2019). Brain-derived neurotrophic factor: a key molecule for memory in the healthy and the pathological brain. Front Cell Neurosci.

[163] Bathina S, Das UN (2015). Brain-derived neurotrophic factor and its clinical implications. Arch Med Sci.

[164] Bauer NM, Moos C, van Horssen J (2012). Myelin basic protein synthesis is regulated by small non-coding RNA 715. EMBO Rep.

[165] Boggs JM (2006). Myelin basic protein: a multifunctional protein. Cell Mol Life Sci.

[166] Strand T, Alling C, Karlsson B, Karlsson I, Winblad B (1984). Brain and plasma proteins in spinal fluid as markers for brain damage and severity of stroke. Stroke.

[167] Can S, Akdur O, Yildirim A, Adam G, Cakir DU, Karaman HI (2015). Myelin basic protein and ischemia modified albumin levels in acute ischemic stroke cases. Pak J Med Sci.

[168] Rymer MM, Akhtar N, Martin C, Summers D (2010). Management of acute ischemic stroke: time is brain. Mo Med.

[169] Musuka TD, Wilton SB, Traboulsi M, Hill MD (2015). Diagnosis and management of acute ischemic stroke: speed is critical. CMAJ.

[170] Cooper D, Jauch E, Flaherty ML (2007). Critical pathways for the management of stroke and intracerebral hemorrhage: a survey of US hospitals. Crit Pathw Cardiol.

[171] (2021). NHLS State Price List 2020/21. National Health Laboratory Service. https://www.nhls.ac.za/diagnostic-services/type-of-tests/.

[172] Lee I, Baxter D, Lee MY, Scherler K, Wang K (2017). The importance of standardization on analyzing circulating RNA. Mol Diagn Ther.

[173] Petti CA, Polage CR, Quinn TC, Ronald AR, Sande MA (2006). Laboratory medicine in Africa: a barrier to effective health care. Clin Infect Dis.

[174] (2021). Lancet: Price Catalogue 2018. https://www.lancet.co.za/wp-content/uploads/2018/08/Uganda-Price-Catalogue-2018-2.pdf.

[175] Lanas F, Seron P (2021). Facing the stroke burden worldwide. Lancet Glob Health.

[176] McLane HC, Berkowitz AL, Patenaude BN (2015). Availability, accessibility, and affordability of neurodiagnostic tests in 37 countries. Neurology.

[177] Drain PK, Hyle EP, Noubary F (2014). Diagnostic point-of-care tests in resource-limited settings. Lancet Infect Dis.

